# Rural opioid treatment program patient perspectives on take-home methadone policy changes during COVID-19: a qualitative thematic analysis

**DOI:** 10.1186/s13722-021-00281-3

**Published:** 2021-12-11

**Authors:** Ximena A. Levander, Kim A. Hoffman, John W. McIlveen, Dennis McCarty, Javier Ponce Terashima, P. Todd Korthuis

**Affiliations:** 1 Division of General Internal Medicine & Geriatrics, Department of Medicine, Addiction Medicine Section, Oregon Health & Science University, 3181 SW Sam Jackson Park Road Mail Code – L475, Portland, OR 97239-3098 USA; 2 Oregon Health & Science University-Portland State University School of Public Health, Portland, OR USA; 3 Oregon Health Authority State Opioid Treatment Authority, Salem, OR USA; 4 Department of Psychiatry, Yale University, New Haven, CT USA

**Keywords:** Substance-related disorder, Addiction medicine, Qualitative research, Methadone, COVID-19, Rural

## Abstract

**Background:**

In the United States, methadone for opioid use disorder (OUD) is highly regulated. Federal agencies announced guidelines in March 2020 allowing for relaxation of take-home methadone dispensing at opioid treatment programs (OTPs) to improve treatment access and reduce COVID-19 transmission risk during the public health emergency. We explored patient perspectives at three OTPs serving rural communities on how take-home policy changes were received and implemented and how these changes impacted their addiction treatment and recovery.

**Methods:**

We completed semi-structured individual qualitative interviews in 2 phases: (1) August–October 2020 and (2) November 2020–January 2021 (total n = 46), anticipating possible policy changes as the pandemic progressed. We interviewed patients with OUD enrolled at 3 rural OTPs in Oregon. Participants received varying take-home methadone allowances following the COVID-19-related policy changes. All interviews were conducted via phone, audio-recorded, and transcribed. We conducted a thematic analysis, iteratively coding transcripts, and deductively and inductively generating codes.

**Results:**

The 46 participants included 50% women and 89% had Medicaid insurance. Three main themes emerged in the analysis, with no differences between study phases: (1) Adapting to changing OTP policies throughout the pandemic; (2) Recognizing the benefits, and occasional struggles, with increased take-home methadone dosing; and (3) Continuing policies and procedures post-pandemic. Participants described fears and anxieties around ongoing methadone access and safety concerns prior to OTP policy changes, but quickly adapted as protocols soon seemed “natural.” The majority of participants acknowledged significant benefits to increased take-homes independent of reducing COVID-19 infection risk including feeling *“*more like a normal person,” improved recovery support, reduced time traveling, and having more time with family and for work. Looking to a post-pandemic future, participants thought some COVID-19-related safety protocols should continue that would reduce risk of other infections, make OTP settings less stressful, and result in more individualized care.

**Conclusions:**

As the pandemic progressed, study participants adapted to rapidly changing OTP policies. Participants noted many unanticipated benefits to increased take-home methadone and other COVID-19 protocols including strengthened self-efficacy and recovery and reduced interpersonal conflict, with limited evidence of diversion. Patient perspectives should inform future policies to better address the ongoing overdose epidemic.

**Supplementary Information:**

The online version contains supplementary material available at 10.1186/s13722-021-00281-3.

## Background

Methadone is one of three highly-effective medications for opioid use disorder (OUD)—significantly reducing morbidity and mortality associated with OUD [1]. Approximately 400,000 patients with OUD in the United States (U.S.) receive methadone [2] which is administered and dispensed following long-established regulatory policies and only available from federally certified and regulated opioid treatment programs (OTPs) [3]. All OTPs follow strict federal regulations [4], with additional state oversight [5]. Newly enrolled patients must present in-person daily (except on Sundays/holidays) to receive their methadone dose. Patients can “earn” take-home methadone doses [6]—medication they self-administer at home—by meeting requirements of (1) duration of continuous treatment engagement and (2) eight criteria intended to assess stability, including documented abstinence of substances, counseling attendance, and housing security [3]. OTPs are not required to extend take-home doses to all eligible patients and payers frequently incentivize continued daily dosing because some provide lower reimbursement for take-home doses [7]. The policies and stigma associated with OTPs are cited as frequent deterrents to patients considering methadone [8, 9]. Those who live in rural communities face high rates of fatal opioid-related overdose and limited availability of addiction treatment services [10], with added challenges to accessing methadone [11]

When the COVID-19 pandemic began in the U.S., some raised concerns about patient and staff safety due to frequently crowded conditions at OTPs [12, 13]. Public health and addiction experts sounded the alarm about the likely synergistic nature of the pandemic and the overdose crisis [14–17]. U.S. overdose mortality rates were already increasing leading up to and through the early part of the pandemic, with concerns these trends would worsen [18]. In response to warnings of increasing severity of the COVID-19 public health emergency, the Substance Abuse and Mental Health Services Administration announced on March 16, 2020 relaxation of federal guidelines around methadone dispensing [19]. These guidelines allowed for, but did not require, states to apply for blanket methadone take-home exemptions of 28 days for “all stable patients” and 14 days for “less stable” patients deemed by OTP staff as able to “safely handle” the amount of medication. The Oregon State Opioid Treatment Authority quickly applied for these blanket exceptions resulting in a dramatic reduction in monthly OTP visits and a 97% increase in number of take-home doses per month [20].

As the pandemic worsened, mandatory lockdowns and social distancing requirements raised substantial worries about the ongoing availability of addiction treatment, support programs, and harm reduction services [21], as well as overall population mental health [22]. Those in rural communities expressed concern about worsening mental health (loneliness, stress, boredom), variability in substance availability, and increased frequency of using substances alone [23]. Little is known about how COVID-19-related changes to OTP policies for take-home methadone affected treatment access and recovery and mental health support for patients in rural communities. We explored patient perceptions on how COVID-19-related OTP policy announcements were received and implemented in rural communities and how these policy changes and the COVID-19 pandemic affected patient addiction treatment and recovery.

## Methods

### Study design

We conducted a qualitative study following the COnsolidated criteria for REporting Qualitative research checklist [24]. Our study was a pre-designated aim of a COVID-19 research supplement to the Oregon HIV/HCV and Opioid Prevention and Engagement (OR-HOPE UH3 DA044831-03S1) study of how OTPs implemented methadone take-home policy changes related to COVID-19 and outcomes of these changes throughout the pandemic. Given the unpredictable time course of the pandemic, we structured the study to examine outcomes over time at pre-designated time points. The Oregon Health and Science University Institutional Review Board (IRB #00021789) approved the study.

### Participants and setting

At the start of the study, Oregon had 20 certified OTPs, mostly located in urban settings. We recruited participants from 3 OTPs in rural Oregon—(1) Adapt in Roseburg with population of 23,000 in southwestern Oregon (2) Adapt in North Bend with population of 9,700 on the southern coast and (3) CODA, Inc. in Seaside with population of 6,700 on the northern coast. Adapt is a non-profit organization that provides primary care, mental health, and addiction treatment services—including adolescent care, women’s tailored services, withdrawal management, office-based opioid treatment (OBOT) services, and two OTPs. At study initiation, Adapt had 255 and 120 patients receiving methadone at their OTPs in Roseburg and in North Bend, respectively. CODA, Inc. is a non-profit organization that offers residential services, OBOT programs, maternal-focused care, and operates two OTPs, one large urban program located in Portland and one OTP in Seaside. The CODA, Inc. Seaside location opened in February 2020, a few weeks prior to COVID-19-related lockdowns and policy changes.

All participants were adults ≥ 18-years-old with OUD, English-speaking, and receiving methadone at study enrollment. All 3 sites reported only enrolling English-speaking patients at the study start. We used convenience sampling for recruitment. Adapt staff recruited participants by chart review, attempting to recruit those in different methadone dosing tiers, then approaching individuals for the study and to coordinate an interview time at the OTP in a private room. CODA, Inc. placed recruitment flyers in their intake window at the Seaside location. Interested participants signed a release of information and study staff contacted them to schedule an interview time with participants at a private location of the participant’s choosing. The recruitment goal was eight different participants from each OTP site for two phases—(1) August–October 2020 and (2) November 2020–January 2021 (total 48 participants) with phases selected to coincide with possible seasonal changes in the pandemic. We enrolled 46 participants (22 in phase 1 and 24 in phase 2) with no participant interviewed twice. All participants approached by Adapt staff enrolled in the study. Three potential participants from CODA, Inc. could not be reached after completing release of information and one enrolled participant was later excluded from analysis after participant disclosed receiving buprenorphine after completing consent.

### Procedures

Research team members (XAL, KAH), addiction medicine health services researchers trained in qualitative research methods, conducted all interviews and had no prior relationship with participants. Study interviewers reached participants via phone to describe the study, obtain verbal consent, and conduct the interview. The study collected demographic information and a single audio-recorded interview with mean interview length of 33 min (SD 8.1). Interviewers used a semi-structured interview guide (Additional file 1) that included questions on (a) COVID-19-related changes to take-home methadone dosing and OTP procedures, (b) benefits and challenges around take-home methadone, (c) concerns for safety around infection and overdose, and (d) policy considerations for post-pandemic. Study participants were compensated for their time with a gift card. Audio recordings were professionally transcribed, and checked for accuracy as needed. Participants were unable to review recordings, transcripts, or study findings for accuracy.

### Analysis

We imported transcripts into Atlas.ti 8.4 for coding and data management, and conducted a thematic analysis [25]. The coding team (XAL, KAH, JPT) developed an initial coding framework after first reading through the transcripts and while referencing the interview guide and study research questions. Data coding occurred in an iterative process consistent with standards for health services qualitative research [26]. One coder (XAL) initially coded three transcripts and conducted coding team meetings after each transcript was coded to resolve discrepancies, reach consensus on coding, and make changes and clarifications to the codebook. XAL and KAH used the refined codebook to dual-code four transcripts to ensure intercoder reliability > 80% [27], with JPT reviewing discrepancies. The remaining transcripts were individually coded (XAL, KAH). The coded data were analyzed for themes consistent with thematic analysis methods [25] with regular coding team meetings to review coding and analysis progress and discuss emerging and salient themes. No new themes emerged consistent with reaching thematic saturation. Salient themes did not differ between the two study phases.

## Results

### Participant characteristics

Of 46 participants, half were female and half male (Table 1). Participants could choose all racial/ethnic identifiers—96% identified as White, 4% as Hispanic/Latinx, and 13% as American Indian/Alaska Native. All participants had insurance with the majority on Medicaid (89%). We asked participants their current methadone dosing regimen at time of interview—the majority (61%) received one to six take-home doses a week. The remaining participants received 13 doses every 2 weeks (19.5%) or 27 doses every month (19.5%). Participants reported recent substance use, with the majority having no past 30-day opioid (89%) or methamphetamine (93%) use (Table 2).

**Table 1:** Study participant characteristics

Demographics	Number (%) n = 46
Mean Age (in years) (SD)	44.1 (12.5)
Gender	
Woman	23 (50%)
Man	23 (50%)
Ethnicity and race (select all that apply)	
Caucasian/White	44 (96%)
Hispanic or Latinx	2 (4%)
American Indian/Alaska Native	6 (13%)
Take home methadone doses	
1 dose every week	17 (37%)
2–6 doses every week	11 (24%)
13 doses every 2 weeks	9 (19.5%)
27 doses every month	9 (19.5%)
Education	
Less than high school graduation	9 (19.5%)
High school graduate/GED	16 (34%)
Some college	14 (30%)
Associate’s degree/Bachelor’s degree/trade school	7 (15%)
Employment status	
Employed	12 (26%)
Unemployed/Looking for work	20 (43%)
Retired	2 (4%)
Disability	11 (24%)
Other (odd jobs/seasonal/temp work)	1 (2%)
Relationship Status	
Married	4 (9%)
Widowed	3 (6%)
Divorced/Separated	6 (13%)
Never Married/Single	20 (43%)
Living with Partner/Partnered	13 (28%)
Homeless in the past 6 months	
Yes	3 (7%)
No	43 (93%)
Insurance (select all that apply)	
Medicaid (Oregon Health Plan)	41 (89%)
Medicare	5 (11%)
Employer-based	3 (7%)
Health Insurance for Tribal Community Members	1 (2%)

Race/ethnicity and insurance percentages do not total 100% as participants could select all applicable choices

**Table 2:** Study participant self-reported substance use

Opioid use other than methadone in the past 30 days	
Yes, non-prescribed	4 (9%)
Yes, prescribed	1 (2%)
No	41 (89%)
Benzodiazepine use in the past 30 days	
Yes, non-prescribed	1 (2%)
Yes, prescribed	2 (5%)
No	43 (93%)
Methamphetamine use in the past 30 days	
Yes	3 (7%)
No	43 (93%)
Alcohol use in the past 30 days	
Yes	5 (11%)
No	41 (89%)

We identified three main perspectives of COVID-19 OTP policy changes around take-home methadone: (1) Adapting to changing OTP policies throughout the pandemic; (2) Recognizing the benefits, and occasional struggles, with increased take-home methadone dosing; and (3) Continuing policies and procedures post-pandemic.

### Adapting to changing OTP policies throughout the pandemic

#### Rapid implementation of early policies

In the lead-up to COVID-19 related lockdowns in March 2020, participants recounted concerns about ongoing methadone access, uncertainty about how OTPs would alter policies, and fears about the nature and severity of the pandemic. Once OTP policy changes were announced, however, participants noted their quick implementation.

…One day it was like boom. Everything changed. They kind of worked us up into things might be changing a little bit…giving you the heads up that this COVID thing is getting kind of serious, but they didn't over-react. They didn't panic… They were just really afraid that this COVID was going to take us all out, you know? – [53-year-old woman, P4]

Participants noted rapid roll-out of extensive safety precautions, which they followed to keep themselves and others safe. One participant stated that “*no employees or no patients have gotten [COVID] yet so I guess either we have gotten lucky or something is working” [49-year-old man, P39].* While inconvenient, participants mostly reported abiding the safety rules.

*Sometimes you don't want to wear a mask, it's suffocating…[but] It's just part of what's going on. If we want to be safe and not catch it, then that's it. I don't want to give nothing to nobody and I don't want to catch nothing. So I'll go with the program…do what I got to do, just to keep safe. [48-year-old man, P2]*

#### Settling into OTP policy changes and new requirements

Participants noted that new policies quickly seemed routine—wearing masks, answering screening questions, and practicing social distancing.

*Now everybody is kind of used to it…secondary motions now—are everyday things. It’s like everybody wearing a mask, it’s like [at the beginning], ‘haha what are you doing?’ and now everybody wears them. It’s just natural now. – [53-year-old woman, P4]*

*…When it first happened, it had us all on edge, but I think that as time goes by and…our daily life adapts to it, I think that we just have a healthy respectful fear of [COVID-19], but we have learned to deal with it. – [41-year-old man, P12]*

#### Rolling back of COVID-19-related increased take-home doses

A few participants from one OTP voiced frustrations about being given increased take-home doses which were rolled back to their prior, pre-COVID, regimen. This policy change happened at the OTP-level around June or July 2020.

*I don’t like [going from one month to 2 weeks] at all but, honestly, you don’t rattle the cage too much…I feel kind of put upon in a way because…I shouldn’t be in there with all the people. I am staying away from the grocery stores and everything but my methadone—of course. Anyway, I am not happy, but I’m not mad either. Just disappointed …They said COVID was over basically, I think COVID’s worse than ever. – [64-year-old woman, P19]*

*Well, personally I think they should have stayed on getting us more take outs… not very long ago they switched back to every day and it seems like the people that were doing good … it seems like they would stay with that because the outbreak isn’t over by any means. – [27-year-old man, P21]*

### Recognizing benefits, and occasional struggles, with take-home methadone dosing

Participants rarely reported challenges with self-managing increased take-homes. They noted using lockboxes—either self-purchased or provided by OTP staff— and following instructions for dosing.

*I really don’t have a challenging part. I thought it went very easy. When I took one I saved the bottle, when I take the next one I save the bottle and I bring those back show them to the nurses…So I wouldn’t even really say there was any difficult or frustrating parts to it to be quite honest.” [27-year-old man, P22]*

Overall, participants recognized how increased take-home doses, and thus coming to the OTP less frequently, had the anticipated outcome of reducing risk of spreading COVID-19. *“Not having to come here [to the OTP], that right there is probably the best protection [from COVID] you can get.” [44-year-old man, P30].* Participants shared additional benefits beyond reducing infection risk by having increased take-homes earlier than they would have under pre-pandemic protocols.

#### Enhanced self-esteem and feelings of normalcy

Receiving increased take-homes, with the added responsibility to manage their medication, resulted in feelings of pride for some participants. These participants valued the trust their OTP was giving them, which gave them more self-confidence.

*I didn’t feel nervous… that I would take them all at once or have trouble taking them every day. I didn’t feel like I wasn’t being monitored properly because I wasn’t coming into the clinic all the time…When you get your take-homes it’s like you feel you are being trusted to take care of yourself, and do the right thing…it felt great…that I was on the right track in my recovery. – [39-year-old woman, P29]*

Participants also valued how increased take-homes, and reduced OTP visits, provided them with a sense of normalcy and stability.

*[I am] able to live a normal life without having to come in every single day. I have a baby at home and stuff so that’s initially why I joined the clinic…Not having to come in. I feel a little more independent. I feel when I do get a job it will be a lot easier…I just enjoy being able to be more like a normal person, just having my medication at home. [31-year-old woman, P15]*

#### Reinforcing and supportive of recovery

For some participants, having more take-homes supported their ongoing recovery through a sense of accomplishment and reward.

*I get all these take-homes and then soon in 53 days, I will get a month worth of take-homes. I will only have to come in once a month and that will be great…I don’t have to stop what I am doing to come in to dose. And it also helps me with my recovery just to get these benefits of take-homes…It makes me feel proud of myself. – [39-year-old woman, P9]*

Other participants found that spending less time in the OTP helped their recovery as staying home allowed them to avoid unstable patients. Seeing “*people that aren’t staying clean and can be nodding out*” was triggering for some participants to “*go get high*” *– [60-year-old woman, P33].*

*Unfortunately, the people who come here…my old people [are] the people you try—necessarily to not spend that much time with anymore…I like to try to stay away as much as possible. I’d rather not see a lot of them if I don’t have to…Especially since I have been doing good for a while, kind of earned it anyways so I felt safe from my sobriety. It worked out good. – [38-year-old man, P35]*

#### Reclaiming time spent traveling doing other rewarding activities

For participants, many of whom lived far from their OTP, increased take-homes significantly reduced time traveling and was helpful for those who “*can’t afford the gas to get [to the OTP] every day*”—[29-year-old woman*, P20].* Participants noticed reduced stress with not needing to *“[get] up an hour earlier every day”* in order to dose before work—* [45-year-old man, P14].*

With the added time, participants shared engaging in rewarding activities. Living in rural communities, many chose to spend time outdoors:

*I was able to go camping with my mom and not have to worry about asking for extra doses. I went and saw my son and I didn't have to ask for extra doses 'cause I already had them. Just made it a little easier. A lot easier. – [51-year-old woman, P18]*

*It gives me a little break. [I can do] other things, like going to the river. I went and floated this weekend, and just hanging out with dad and barbecuing and doing yard work and stuff like that. – [48-year-old man, P2]*

Participants with children, particularly women, noted significant benefits to increased take-homes – not needing to arrange child care and having more time for family.

*I am a single mom and…especially now that I am back to work, it is nice and convenient for me because I have to be to work at a certain time and it's hard for me to guarantee that I can get in [to the OTP] as often…it's definitely made it easier. – [33-year-old woman, P11]*

#### Struggles with take-home methadone

While most participants expressed success with increased take-homes, a few participants acknowledged challenges others may have experienced. “*I know from talking about it in group, some people can feel overwhelmed with all the take-homes and some people don't trust themselves.” – [38-year-old woman, P29].*

A couple of participants expressed personal concerns around receiving increased take-homes including “*start[ing] to feel almost complacent in a sense, that there wasn't any effort that I needed to put into obtaining my medication” [29-year-old man, P10]* or being too early in methadone treatment.

*Now, I like coming in everyday because I think it keeps you on track…I think it's better for people at first…I wasn't even getting take-homes, and all of a sudden here I am getting two weeks of my medicine so it was kind of a lot…For me it just wasn't good at the time because I was still pretty new in my sobriety, you have to trust in yourself and everybody is different. – [44-year-old woman, P25]*

### Continuing policies and procedures post-pandemic

OTPs nationwide made numerous COVID-19 related policy modifications including increased take-homes, workflow improvements, social distancing, and disinfecting protocols [28]. These changes were intended to prevent COVID-19 infection—to keep patients and staff safe. Participants were asked to provide their perspectives on what clinic policies and changes might be helpful to maintain after the pandemic passed.

#### COVID-19 protocols could prevent spreading other infections

Clinics implemented changes to minimize viral spread including plexiglass shields, limiting numbers of individuals in waiting rooms, increased sanitation procedures, and required face masks. Overall, participants thought maintaining some of these safety measures after the pandemic passes would be wise, given they also prevented other infections.

*I think that they still should have the glass shields…I mean, [it’s] not just COVID, you can still get the flu from people. For somebody like me, getting the common cold can be-- that's what I went to the hospital for, my grandson gave me the rhinovirus which is the common cold. So, it's not just COVID. – [49-year-old woman, P44]*

This, in turn, meant they were less likely to spread infections to others, particularly family members and children.

*The fact that it's limited amount of people, not so crowded in here, you know? So I feel a lot more comfortable sitting in here waiting when it's not as many people and for the fact of not spreading any diseases or colds. – [29-year-old woman, P20]*

#### Social distancing created a more supportive environment for recovery and mental health.

Participants also noted unintended positive effects of social distancing – less people in the waiting room –reduced crowding created a healthier mental health atmosphere.

*I like how it's not as crowded…That's nice because when there [are] too many people in the waiting room, it triggers anxiety and it does a lot on the head. PTSD sort of type thing when everybody gets loud…So now…it's usually pretty quiet and not hectic. – [29-year-old man, P16]*

Some participants noted that less intermingling had reduced confrontations and would be a worthwhile policy to maintain beyond the pandemic.

*[I would continue] the social distancing, just making sure that there's not a lot of people in the waiting areas. It gets crowded and people are standing and people that don't get along here so it causes more problems… [this is] a small, community… a lot of people know a lot of people -- some people that go here that are still in the addiction…you always see someone that you know or that you have a problem with. So a lot of fights seem to happen. – [31-year-old woman, P3]*

A few participants noted how social distancing, particularly requiring people wait outside, might need reexamining during inclement weather conditions. Waiting room capacity limits would, at times, necessitate clients queuing outside. Participants noted that other workflow streamlining were generally moving patients more quickly through daily dosing procedures—*“in and out”*—which was an improvement “*that works out for everybody”— [53-year-old woman, P4].*

#### Desiring of more tailored, individualized OTP services

With many participants experiencing added benefits of take-homes, but a few needing or wanting more support, the COVID-19 pandemic highlighted to participants the previous inflexibility of federal guidelines. Some participants expressed hope for more individualized care in the future.

*I would like to see is maybe not having to come in quite as often…Everybody is different. Everybody should be looked at on an individual basis…maybe the people who make their appointments, the people who are making an effort to make a change, and that are certainly trying to utilize the program…I would hope for…less having to come in just to dose. I don't mind taking seven days at a time. I was okay with the Monday, Wednesday and Friday. I thought that was very fair because I was still able to have contact with counselors…even if I didn't have an appointment. – [27-year-old man, P22]*

## Discussion

Our study describes experiences of 46 patients from rural Oregon communities receiving varying amounts of take-home methadone during the COVID-19 pandemic. Participants shared how they adapted to rapidly changing rules and policies; benefitted, and rarely struggled with take-home methadone; and also recognized opportunities for future policies that may improve their care. These findings suggest ways that OTP policies can be modified post-pandemic to be more patient-centered [7], particularly for those living in rural communities [29].

The COVID-19 pandemic has offered a critical opportunity to reevaluate and reprioritize addiction treatment to better address the ongoing overdose epidemic. [30, 31]. Signals of ongoing high rates of overdose-related mortality continued throughout 2020 [32, 33]. Restructuring U.S. state and federal OTP regulatory policies and methadone regulations [34, 35], and considering alignment with policies in other countries [36, 37], are key components to improving treatment access.

Pharmacist-prescribed and community pharmacy-dispensed methadone, as is already efficaciously done in Canada, Australia, and many countries in the European Union, is one emerging policy consideration in the United States [38, 39]. Those living in rural communities face significant barriers and limited access to addiction services, especially methadone [10, 40]. Several participants in our study shared how spending less time driving and more time doing more rewarding activities, and saving money on fuel, were major benefits to increased take-home doses. These findings are consistent with other studies evaluating barriers to addiction treatment faced by those living in rural areas [11]. Policy changes that allowed for pharmacy-dispensed methadone could significantly increase rural methadone access [38, 41], and could make the U.S. addiction treatment infrastructure more resilient during future public health emergencies.

Most participants in our study reported little difficulty with self-managing their increased take-home methadone doses—keeping bottles secured in lockboxes and taking doses as instructed. Being entrusted by OTP staff with this added responsibility also provided a sense of self-worth and accomplishment. Given study participant responses to take-home methadone, policymakers should reconsider criteria for determination of patient stability and long timelines for earning increased take-home doses used pre-pandemic [42]. Concerns around diversion or methadone-related overdoses motivate maintaining the status quo [43], and have been expressed by OTP staff and leadership during the pandemic [28, 44]. Alternatives to physically presenting to OTPs to dose include technology-assisted dosing via tele-monitoring or automated home medication dispensers [45, 46]. Mobile delivery of methadone is another viable option [47], particularly with recent Drug Enforcement Administration (DEA) announcements allowing for mobile vans operated by DEA-registered OTPs [48]. A couple participants mentioned challenges with increased take-homes, and appreciated returning to daily dosing. Future OTP policies should consider more flexibility and individualization of treatment plans.

Participants described how taking their methadone at home improved their ability to work and to spend time with their family—providing them with a sense of a “normal life.” They also expressed how spending less time at the OTP and, when there, in waiting rooms that were less crowded, supported their overall mental health and recovery. These findings align with research on patient preferences for buprenorphine over methadone [8]. Many patients, however, may prefer methadone or find it more effective than buprenorphine [49]. Thus, strict OTP policies may limit access to a highly-effective treatment. Receiving methadone in an office-based setting would be more patient-centered [50] and pilot studies transferring stable patients from OTPs to primary care settings for ongoing methadone have been successful [51].

Our study has limitations—given the qualitative approach, we cannot make projections of results beyond this study population [52]. Participants in our study self-reported rare use of substances outside their prescribed methadone, and thus likely represent a more stable population. Efforts should be made to explore patient perspectives to take-home methadone during the COVID-19 pandemic in patients less stable or newer to methadone. Findings from Connecticut, however, did not show an increased rate of methadone-related overdose during the pandemic [53] and a survey of patients in North Carolina at OTPs reported rare instances of methadone diversion [54]. Our study also lacked racial and ethnic diversity, and while consistent with the demographics of rural Oregon, limits generalizability to more diverse communities. Understanding and addressing barriers to addiction treatment, including methadone, in minoritized and structurally vulnerable communities is critical with rising overdose rates in these populations [55, 56]. Methadone regulations in the United States have a racialized history and take a carceral approach to treating addiction [57]. Given the ongoing racial and ethnic disparities in accessibility and utilization of methadone compared to buprenorphine [58–60], understanding the experiences and implementing the treatment preferences of these communities is necessary to provide equitable and just care.

## Conclusion

Our study provides important and needed insights into patient perspectives of OTP-related policy changes during the COVID-19 pandemic. Patients with OUD receiving methadone in rural communities adapted to these rapidly changing events and noted many benefits that should inform future policy development post-pandemic to better address the ongoing overdose epidemic.

## Supplementary Information

**Additional file 1.** Semi-structured Interview Guide.

## Data Availability

The datasets used and analyzed during the current study are available from the corresponding author on reasonable request.

## Abbreviations

DEA: Drug Enforcement Administration
OBOT: Office-Based Opioid Treatment
OTP: Opioid treatment program
OUD: Opioid use disorder
U.S.: United States
