# Supplementary material for: Rural opioid treatment program patient perspectives on take-home methadone policy changes during COVID-19: a qualitative thematic analysis
Source: Addict Sci Clin Pract. 2021 Dec 11;16:72. doi: 10.1186/s13722-021-00281-3 (PMC8665717; doi:10.1186/s13722-021-00281-3)
Supplement: Supplementary file 1 — Additional file 1. Semi-structured Interview Guide. [file 13722_2021_281_MOESM1_ESM.docx]

Online Supplement – Semi-structured Interview Guide

Thank you so much for talking with me today. We are interested in understanding how the COVID-19 changes in clinic operations have affected you. Everything you tell me will be kept confidential and we won’t share your name with anyone besides study staff. We record interviews to help with remembering the information you share. Stop me at any time if you have any questions, if anything is unclear, or if you would prefer to skip a question.

Your participation may help to make things better for people in your situation and those who come along after you, so we appreciate the time that you are taking to talk to us.

What questions do you have before we begin?

OK, I’ll turn the recorder on now and we’ll get started.

*Turn on recorder: State today’s date, the current time, and the Participant ID.*

Example: “Today is August 2nd, it is 11:30am, and I’m talking to Participant 112.”

Pre-COVID Patient OTP Information

1. When did you enroll in the __________ clinic? Date: ____________________
2. Around March 1, 2020 how often did you come to the clinic for methadone: ___________________ and for group or individual counseling: ___________________________

Post-COVID Patient OTP Information

For the rest of the interview we will be focusing on changes that occurred with your treatment at [OTP] after COVID-19 related changes that began in mid-March, 2020.

1. At the end of March how often did you come to the clinic for methadone: ___________________ and for individual or group counseling: ___________________________?
2. What were you told about the reasons for changing how often you needed to come to clinic for your methadone?
3. How did you hear about these changes?
4. What changes would you want to see continue after the COVID-19 crisis passes?

Picking up Medication Doses during COVID

1. About how long do you usually have to wait in-line to receive your methadone?

How does this compare with waits before COVID?

1. About how many other people are usually waiting when you’re there? How possible is it to stay six feet apart from other people?
2. What precautions are you taking when picking up your methadone? What precautions are staff taking?
3. How well do think this is working to keep people from getting COVID-19?
4. What could be done to make people feel more protected from getting COVID-19?

Experiences with Take Home Methadone

1. What has your experience been with take-home methadone so far?
2. How has the frequency of clinic visits changed? For example, are you coming in less often or more often?
3. How did increased access to take-home doses affect you?

Potential probes: What has been beneficial/helpful about having more doses? What has been challenging or difficult about it?

1. How have you stored your methadone? Have you had any issues with the methadone being lost or stolen?
2. Have you ever used more than one dose of your methadone per day? Have you ever split your dose during the day? If so, why? Please tell me about a time since mid-March when you took more or less of your methadone than you were told to take.
3. How frequently do you have urine screens? Is this a change for you? Why has this changed?

Interplay of COVID and SUD / COVID and Mental Health

1. How has COVID-19 affected your mental health and well-being?
2. Probes: What counseling or treatment for mental health have you received during COVID-19?
3. How do you participate in [self-help groups/peer support] during COVID-19—for example, in-person, phone call, online?
4. How has your OTP clinic conducted individual counseling sessions during COVID?
5. How has your OTP clinic conducted group sessions during COVID?
6. [If not already addressed in Q17 and Q18] What role has telemedicine had in your care since COVID started? (Define if needed - including visits by telephone or by audio/video) What access do you have to reliable Internet? How does it compare with in-person counseling or group visits?
7. Do you have concerns about your risk of overdose during COVID-19? [if yes] What are those concerns? Do you have concerns about your risk of return to use during COVID-19? [if yes] What are those concerns?
8. How, if at all, has COVID-19 affected the risk of overdose for you or others you know? How, if at all, has COVID-19 affected the risk of return to use for you or others you know?
9. How, if at all, have the things you do to stay safe from overdose changed during COVID-19?
10. Do you currently have Narcan/naloxone? [If yes] Where did you get it?

Psychosocial Factors

1. Could you please tell me about where you live right now?

a. How long have you been there?

b. What concerns do you have about the risk of getting COVID-19 through your housing situation?

c. What challenges do you face in limiting the number of people you’re exposed to related to your housing situation? (For example: couch-surfing, moving to different peoples’ houses, living in camp or shelter, living in a house with lots of people around.)

d. What, if anything, has changed during COVID-19 about who you’re spending time with or how many people you have around?

1. Could you please tell me about what you use for transportation?

a. Any other ways you get around?

b. What concerns do you have about the risk of getting COVID-19 through your transportation?

c. What challenges do you face in limiting the number of people you’re exposed to related to your transportation? (For example: relying on others for rides, other people relying on you for rides, buses or ride share.)

1. Could you please tell me about how you earn income?

a. How might this affect your risk of getting COVID-19? (For example: bottle or can exchange, close interaction with others.)

b. What, if any, changes have you had in your income due to the requirements to remain at home?

1. How worried are you about getting a COVID-19 infection?

1. How do you try to keep safe?
2. Is there anything else you would like to share?

Thank you for your time.
